# *Spiradiclis tubiflora* (Rubiaceae), a new cave-dwelling species from southern China

**DOI:** 10.3897/phytokeys.130.34625

**Published:** 2019-08-29

**Authors:** Lei Wu, Bing-Mou Wang, Bo Pan, Xun-Lin Yu

**Affiliations:** 1 College of Forestry, Central South University of Forestry and Technology, Changsha 410004, China Central South University of Forestry and Technology Changsha China; 2 Panyu Central Hospital, Guangzhou, 511400, China Panyu Central Hospital Guangzhou China; 3 Guangxi Institute of Botany, Guangxi Zhuang Autonomous Region and the Chinese Academy of Sciences, Guilin 541006, Guangxi, China Guangxi Institute of Botany, Guangxi Zhuang Autonomous Region and the Chinese Academy of Sciences Guilin China

**Keywords:** Rubiaceae, taxonomy, Guangdong, limestone

## Abstract

*Spiradiclis tubiflora*, a new Rubiaceae species, is described from a limestone area of southern China. It is similar to *Spiradiclis glandulosa* and *S. umbelliformis*, but differs from the latter two in its linear stipule, short peduncle and tubular-funnelform corolla with a distinctively enlarged tube. The colour photograph, illustrations, detailed descriptions and conservation status of the new species are provided.

## Introduction

Caves are considered to be extreme and exceptional habitats that usually provide insufficient resources, especially lack of light, water and soil for plants to survive (Whitten 2009). Most caves are isolated environments which lead to the limitation in dispersal or movement of species and provide great possibilities for speciation and radiation (Biswas 2009, Chung et al. 2013). Many cave-dwelling species, especially those from karst areas, are highly localised (Chen 2006, Whitten 2009). Recently, the number of newly discovered and described plant species with unique characters from China’s karst caves is increasing dramatically, including *Begonia* L. (Begoniaceae, e.g. Peng et al. 2012), *Chiritopsis* W. T. Wang (Gesneriaceae, e.g. Wu et al. 2011), *Elatostema* J.R.Forster & G.Forster (Urticaceae, e.g. Fu et al. 2017), *Lagarosolen* W. T. Wang (Gesneriaceae, e.g. Xu et al. 2011), *Pilea* Lindl. (Urticaceae, e.g. Monro et al. 2012) and *Polystichum* Roth (Dryopteridaceae, e.g. Han et al. 2016). *Spiradiclis* Blume also exhibits a great diversity in cave habitat with six newly published species from karst caves (Deng et al. 2014, Wen et al. 2015, Wu et al. 2015a, 2015b, 2016, Liu et al. 2018).

There are approximately 53 *Spiradiclis* species worldwide, most representatives being herbs and occurring in limestone areas (Chen and Taylor 2011, Deng et al. 2014, Wang et al. 2015, Wen et al. 2015, Wu et al. 2015a, 2015b, 2016, 2019, Wang 2016a, 2016b, Pan et al. 2016, 2019, Liu et al. 2018). China is the diversity centre of *Spiradiclis* with 47 species being recorded and most of them distributed in Guangxi and Yunnan provinces of south-western China (Lo 1999, Chen and Taylor 2011, Liu et al. 2018, Pan et al. 2019).

*Spiradiclis* is a taxonomically difficult genus and most similar to *Ophiorrhiza* L. (Chen and Taylor 2011), some specimens of *Spiradiclis* and *Ophiorrhiza* with flowers have even been frequently misidentified with each other (Wu et al. 2015a). However, *Spiradiclis* can be distinguished from *Ophiorrhiza* by its linear-oblong or subglobose capsules with two or four valves when mature (vs. obcordate and compressed capsules with two valves when mature) (Lo et al. 1983, Robbrecht 1988, Lo 1999, Chen and Taylor 2011). The genus was split into two subgenera: subgenus Spiradiclis characterised by ellipsoid to linear-oblong capsules with twisted valves when mature and subgenus Sinospiradiclis H.S.Lo characterised by subglobose capsules with untwisted valves (Lo 1998).

**Figure 1.**
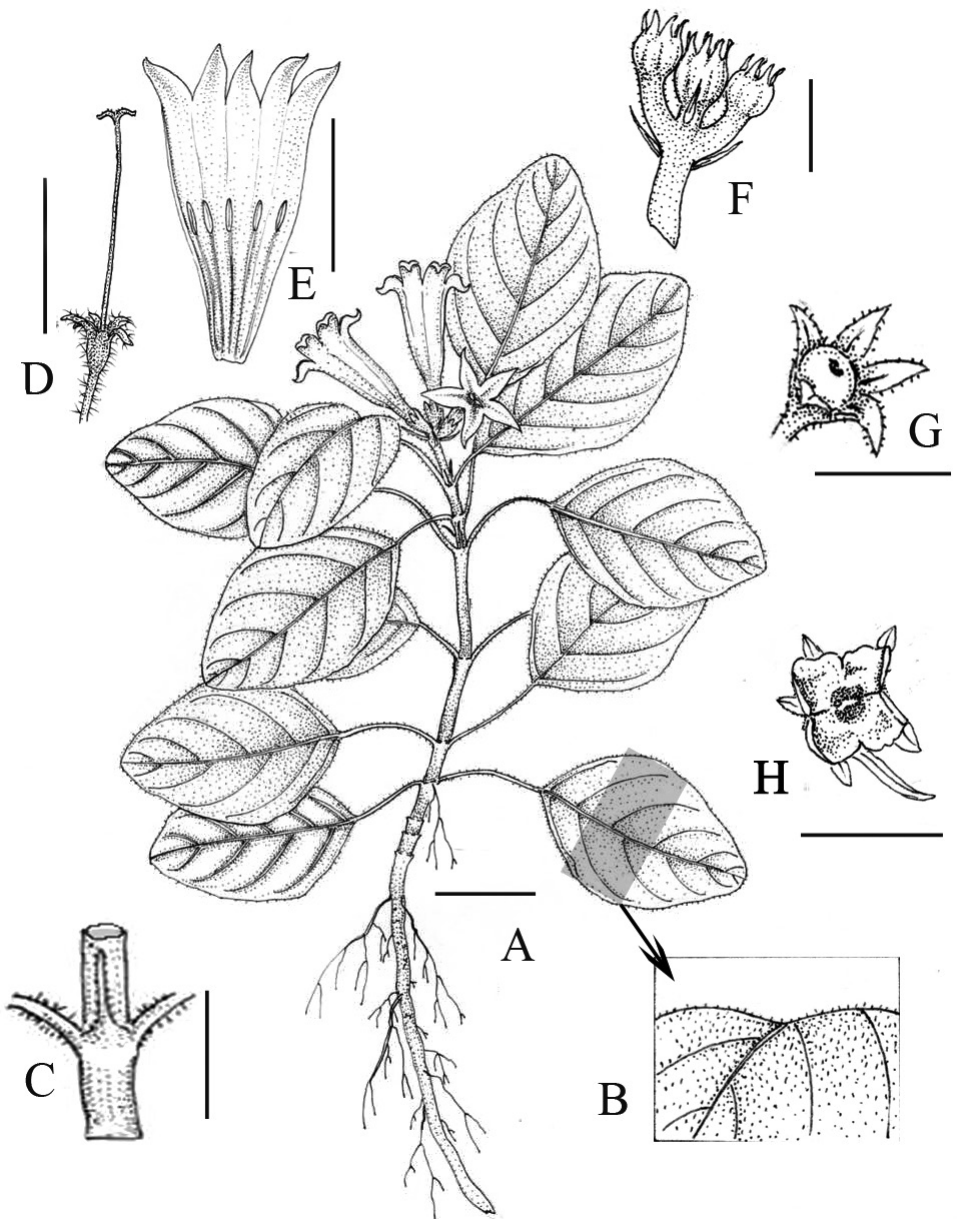
*Spiradiclis tubiflora*. **A** Habit **B** Enlarged leaf blade (adaxial) **C** Stipule **D** Style **E** Opened corolla **F** Infructescence, lateral view **G** Capsule before dehiscence **H** Matured capsules split into four valves. Scale bars: 1 cm (**A, D, E**); 3 mm (**C, F–H**). Drawn from the holotype by Zheng-Meng Yang.

**Figure 2.**
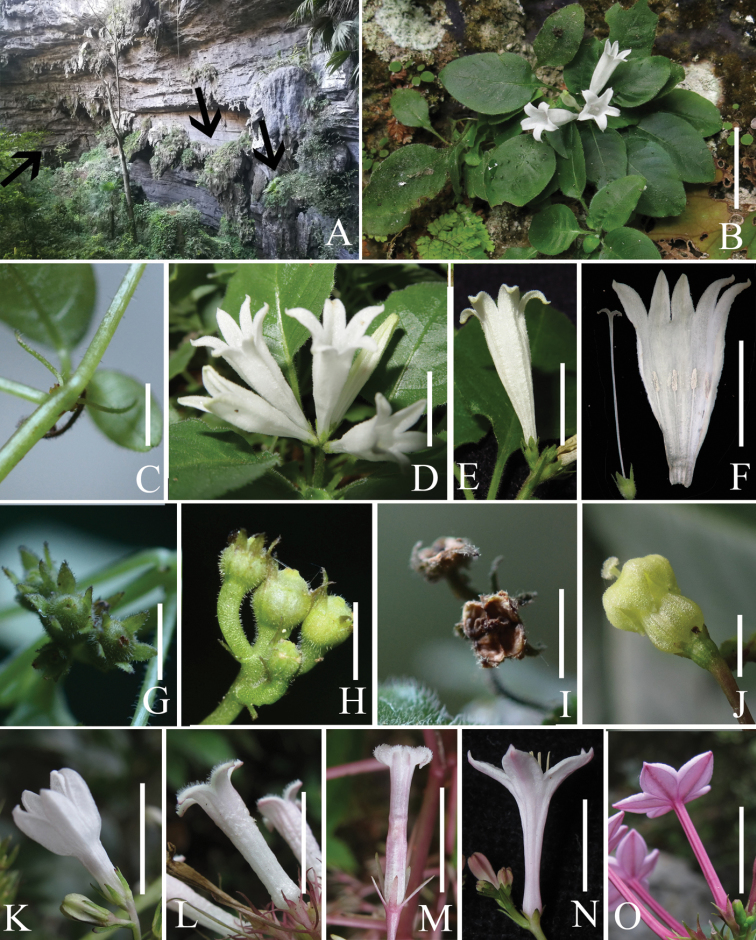
*Spiradiclis tubiflora*. **A** Habitat (the arrow shows the place of growth) **B** Habit **C** Stipule **D** Inflorescence, lateral view **E** Flower, lateral view **F** Corolla opened to show floral parts **G** Capsules, frontal view **H** Capsules, lateral view **I** Matured capsules with four valves. Flowers of selected Spiradiclis species, lateral views: **J***S. longipedunculata***K***S. fusca***L***S. malipoensis***M***S. baishaiensis***N***S. glabra***O***S. coccinea*. Scale bars: 1 cm (**B, D, E, F, K, L, M, N, O**); 3 mm (**C, G, H, I, J**). Photos by Bo Pan, Jing Liu and Lei Wu.

During a field investigation of the karst cave in Guangdong Province, southern China in 2009, a peculiar species of Rubiaceae was found. The plant has subglobose capsules, dehisces with four valves and many small, granulate seeds when mature (Figs 1F, 2I) which clearly indicated it belongs to *Spiradiclis*. After re-collections of flowers and fruit materials and further comparison of the known *Spiradiclis* species, we confirmed that it is an unpublished species and report it here.

## Material and methods

Materials are deposited at the herbarium of forest plants in Central South University of Forestry and Technology (CSFI), Guangxi Institute of Botany, Guangxi Zhuang Autonomous Region and Chinese Academy of Sciences (IBK). Morphological observations and measurements of the new species are based on living material in the field, as well as dry specimens. The conservation status of the new species is evaluated, based on field observations in accordance with IUCN guidelines (2016).

## Taxonomic treatment

### Spiradiclis tubiflora

Taxon classificationPlantaeGentianalesRubiaceae

L.Wu, B.M.Wang & B.Pan sp. nov.

81839A35C8B25FB2830FDE3A39D30E51

urn:lsid:ipni.org:names:60479355-2

Figs 1 , 2A–I

#### Diagnosis.

This species is similar to *Spiradiclis glandulosa* and *S. umbelliformis* by having procumbent to creeping habit. It is, however, easily distinguished from the latter two by its linear stipule, short peduncle and tubular-funnelform corolla with distinctively enlarged tube.

#### Type.

China. guangdong: Yingde city, Jiulong town, on the wall near the mouth of a karst cave, 460 m alt., 24°17'N, 112°36'E, 18 Oct 2016 (flower), B. Pan GXIBPB2016023 (holotype: IBK!; isotype: CSFI!)

#### Description.

Herbs to 5 cm in height, perennial, procumbent to creeping; stems glabrous or subglabrous. Petiole 0.3–1.8 cm, sparsely pubescent; leaf blade drying papery, adaxially green, abaxially pale, ovate to elliptic, 4.5–25.5 × 4.0–14.5 mm, both surfaces pubescent, abaxially with densely yellow glandule-like spots, base rounded to obtuse, sometimes decurrent, apex acute to rounded; secondary veins 3–5 pairs; stipules usually caduceus, pubescent, narrowly linear, 3–5 mm long. Inflorescences cymose, umbelliform to subcapitate, 2–5-flowered; peduncles 1.2–1.5 cm long, densely pubescent; bracts subulate, densely pubescent, 1.8–3.0 mm long; pedicels 1.5–2.0 mm long. Calyx densely pubescent; hypanthium portion subglobose, 1.4–1.6 mm long; lobes triangular, 1.4–1.6 mm long. Corolla white, tubular-funnelform, subglabrous outside; tube 14–16 mm long, ca. 2 mm in diameter at the base, while 3.8–4.5 mm in diameter at the middle of corolla tube, inside densely pubescent near base; lobes ovate to ovate-triangular, 3.5–4.5 × 2.5–3.0 mm. Stamens 5, inserted at the middle of corolla tube; filaments 0.5–1.5 mm long; anthers dorsifixed, linear. Ovary 2-celled, ovules numerous in each cell on peltate axile placentas, attached to the middle of the septum; stigmas 2-lobed, appearing near the throat of corolla tube. Capsules subglobose, ca. 2 mm in diam., densely pubescent, valves 4. Seeds many, dark brown, granular, 0.22–0.28 mm long.

#### Distribution and ecology.

The new species is only known from the type locality. Plants on the wall or large stones inside or at the mouth of the cave, usually wet and covered with calcareous soil. Flowering from August to October, occasional few individuals in March to May, fruiting from September to December.

#### Etymology.

The specific epithet refers to the corolla shape of the new species. The Chinese name is given as “cu-tong-luo-xu-cao (粗筒螺序草)”.

#### Preliminary conservation status.

Up to now, only one population with 360 individuals have been found in the type locality. Although five field investigations have been carried out in the surrounding area of the type locality in the past five years since the new species was discovered, no additional populations have been found. The individuals are occurring in places with thick calcareous soil and thriving in low-light conditions. Karst caves are known for their spectacular landscape and nature which attract tourists. Many karst caves in China played an important role in stimulating the local economy and were exploited for tourism. The cave where the new species occurred has not been spared and the cement road has been built directly leading into the cave, despite this cave being located far away from human settlements. According to the IUCN (2016) Red List Categories and Criteria, *Spiradiclis tubiflora* should be assigned as Critically Endangered (B2ab(iii,iv,v) & D).

#### Discussion.

The corolla character of *Spiradiclis* shows great diversity (Fig. 2J–O). The corolla tube of the genus ranges from 2.5 to 24 mm (Chen and Taylor 2011), the corolla colour appears in white, pink or purple-reddish and the corolla shape varies from urceolate-tubular (*Spiradiclis longipedunculata* S. Y. Liu & S. J. Wei, Fig. 2J), tubular (*S. malipoensis* H. S. Lo, Fig. 2L and *S. baishaiensis* X. X. Chen & W. L. Sha, Fig. 2M), funnelform (*S. fusca* H. S. Lo, Fig. 2K and *S. glabra* L. Wu & Q. R. Liu, Fig. 2N) to salverform (*S. coccinea* H. S. Lo, Fig. 2O). Although the corolla shape of *Spiradiclis tubiflora* is tubular-funnelform, its corolla tube enlarges distinctly from near the base to the throat of the corolla which currently is unique in all the known *Spiradiclis* species (Fig. 2D, E). *Spiradiclis tubiflora* is morphologically most similar to *S. glandulosa* L. Wu & Q. R. Liu and *S. umbelliformis* H. S. Lo by having procumbent to creeping habit, but it can be distinguished from the latter two species (Table 1). According to Lo (1998), this new species belongs to subg. Sinospiradiclis on the basis of its subglobose capsules with four untwisted valves (Figs 1E–G, 2G–I).

Based on our field investigations of *Spiradiclis* in China and careful studies of relevant literature and specimens, about 94% of the known *Spiradiclis* species are confirmed to be distylous plant, of which more than 30 species have been observed with both long- and short-styled flowers in the same population. It is known that being distylous is a unique phenotype in plants to ensure reproduction by avoiding self-pollination and to increase male fitness and outcrossing rates by reducing sexual interference between male and female functions (Watanabe et al. 2017). In this study, however, all the individuals with observed flowers are with stamens inserted at the middle of the corolla tube and stigmas located near the throat of the corolla tube, this being the long-styled form. This phenomenon could be interpreted from two aspects currently: 1) both styled flowers are present in the population but only few individuals are short-styled rather than long-styled and they could be overlooked during the investigations; 2) this population is a monomorphic population with all individuals having long-styled flowers which is not rare and was reported from other groups such as *Eichhornia* Kunth and *Luculia* Sweet (Barrett 1989, Zhou and Wang 2009). Further studies on the reproductive and pollination biology of the new species are needed.

#### Specimens examined

**(Paratypes). CHINA. Guangdong**: the type locality, 27 Nov 2016 (fruit), L. Wu & B. M. Wang 5610 (CSFI!), 13 Oct 2017, L. Wu & B. M. Wang 6236 (CSFI!).

**Table 1.** Morphological comparison of *Spiradiclis tubiflora*, *S. glandulosa* and *S. umbelliformis*.

	*Spiradiclis tubiflora*	*S. glandulosa*	*S. umbelliformis*
Stipule	narrowly linear, usually caduceus	deeply 2-parted, persistent	deeply 2-parted, persistent
Peduncle	1.2–1.5 cm long	2–5 cm long	2–7 cm long
Calyx lobe	triangular, 1.4–1.6 mm long	oblong-lanceolate, ca. 4–6 mm long	ovate-triangular, ca. 0.6 mm long
Corolla	tubular-funnelform	funnelform	funnelform to tubular-funnelform
Corolla tube	14–16 mm long, enlarged distinctly, 3.8–4.5 mm in diam. at middle	16–18 mm long, slender, ca. 1.8 mm in diam. at middle	17–18 mm long, slender, ca. 1.5 mm in diam. at middle

## Supplementary Material

XML Treatment for Spiradiclis tubiflora
